# Enhancing Devulcanizing Degree and Efficiency of Reclaimed Rubber by Using Alcoholic Amines as the Devulcanizing Agent in Low-Temperature Mechano–Chemical Process

**DOI:** 10.3390/polym16030395

**Published:** 2024-01-31

**Authors:** Lei Guo, Lichen Bai, Jinyang Zhao, Kexin Liu, Xingao Jian, Hailin Chai, Fumin Liu, Shouyun Guo, Gongxu Liu, Haichao Liu

**Affiliations:** 1 College of Electromechanical Engineering, Qingdao University of Science & Technology, Qingdao 266061, China; guolei-stirc@qust.edu.cn (L.G.); 15684195159@163.com (L.B.); 15725829737@163.com (J.Z.); 19862125309@163.com (K.L.); jian18765479242@163.com (X.J.); 17806249607@163.com (H.C.); liufumin@qust.edu.cn (F.L.); assadakorn_sk132@hotmail.co.th (S.G.); l18765487207@163.com (G.L.); 2 Sino-Thai International Rubber College, Qingdao University of Science & Technology, Qingdao 266061, China; 3 National Engineering Research Center of Advanced Tire Equipment and Key Materials, Qingdao University of Science & Technology, Qingdao 266061, China

**Keywords:** waste rubber, mechano–chemical reclamation, alcoholic amine, devulcanizing degree, devulcanizing efficiency

## Abstract

Low-temperature mechanical chemical devulcanization is a process that can produce reclaimed rubber with exceptional mechanical properties. However, the inadequacy and low efficiency of the devulcanization have significantly restricted its application. To address the issues, alcoholic amines, including hydroxyethyl ethylenediamine (AEEA), ethanolamine (ETA), and diethanol amine (DEA), are utilized as devulcanizing agents to promote the devulcanization process. Careful characterizations are conducted to reveal the devulcanizing mechanism and to depict the performances of reclaimed rubbers. Results show that the amine groups in the devulcanizing agents can react with sulfur after the crosslink bonds are broken by mechanical shear force, thus blocking the activity of sulfur and introducing hydroxyl groups into the rubber chains. The incorporation of alcoholic amines can enhance the devulcanizing degree and devulcanizing efficiency, reduce the Mooney viscosity, and improve the mechanical and anti-aging performance. When using DEA as the devulcanizing agent, the sol content of reclaimed rubber increases from 13.1% to 22.4%, the devulcanization ratio increases from 82.1% to 89.0%, the Mooney viscosity decreases from 135.5 to 83.6, the tensile strength improves from 14.7 MPa to 16.3 MPa, the retention rate of tensile strength raises from 55.2% to 82.6% after aging for 72 h, while the devulcanization time is shortened from 21 min to 9.5 min, compared with that without using alcoholic amines. Therefore, alcoholic amines exhibit remarkable advantages in the devulcanization of waste rubber, thus indicating a promising direction for the advancement of research in the area of waste rubber reclamation.

## 1. Introduction

As global car ownership continues to rise, the issue of proper disposal and recycling of scrap tires has become increasingly urgent [1,2]. It is estimated that approximately 100 million tires are discarded annually, with less than 10% being reasonably treated and recycled [3], which has resulted in significant environmental pollution and resource waste [4]. One effective method for recycling scrap rubber is devulcanization, which breaks down the crosslinked network structure of scrap rubber and transforms its molecular structure back into a linear form, enabling the high-value reuse of waste rubber [5].

High-temperature dynamic devulcanization [6] is the most commonly used method for the industrial production of reclaimed rubber (RR). The process involves the use of oil and high temperatures (up to 280 °C) to achieve the devulcanization of rubber through swelling and oxidative cleavage [7]. This method is an intermittent process, with each devulcanization production taking approximately 4 to 6 h. Additionally, high-temperature oxidative pyrolysis results in both crosslink bond scissions and main chain scissions, thus greatly impacting the performance of RRs; for instance, the tensile strength is typically below 15 MPa [8].

To enhance the properties of RRs, various methods have been developed, including the thermo–mechanical method [9,10], mechano–chemical method [11,12], microwave method [13,14], ultrasonic method [15,16], and chemical method [17,18]. Among the various techniques, the mechano–chemical method is considered to be the most promising due to its capability of selectively breaking crosslink bonds while preserving the main chain structure of rubber molecules. Scholars have utilized different mechanical devices, including open mills [19], mixing machines [20], twin-screw extruders [21], etc., to achieve the mechano–chemical reclamation of scrap rubber. In our previous work, we proposed a low-temperature rubber reclaiming equipment (LTRE) that combines the advantages of mixing machines and extruders and exhibits excellent devulcanization capabilities [22,23]. The prepared RRs exhibit a remarkable tensile strength ranging from 16 to 18 MPa, significantly higher than that of the high-temperature dynamic method. However, the devulcanizing degree and efficiency of this method are generally lower than that of the high-temperature dynamic method due to the low-temperature condition. The shortcomings of low-temperature techniques have also been confirmed in the studies of Gagol et al. [24] and Shi et al. [25].

Devulcanizing agents play a crucial role in promoting crosslink bond breakage and closing end-group activity, simultaneously affecting the devulcanizing degree and efficiency. Disulfides are the most commonly studied chemicals for RR devulcanization. For instance, Ghosh et al. used bis(3-triethoxysilyl propyl) tetrasulfide (TESPT) to reclaim waste SBR, and it not only promotes the devulcanization but also facilitates the dispersion of silica in the nonpolar SBR matrix [26]. Azarabtin et al. [27] compared the devulcanizing effects of tetramethylthiuram disulfide (TMTD) and disulfide oil on waste rubber and found that TMTD performed better than disulfide oil. Amines are also effective for the mechano–chemical devulcanization process [28]. Kojima et al. [29] used thiophenol (PhSH)-n-butylamine (n-BuNH2) as the devulcanizing agent to reclaim unfilled polyisoprene rubber under the action of supercritical CO_2_, resulting in 100% soluble products. Molanorouzi and Mohaved [16] used hexadecyl amine as the devulcanizing agent to reclaim waste tire rubber under microwave irradiation and proposed an ionic devulcanization mechanism based on amines. Jiang et al. [30] used tetraethylenepentamine (TEPA) as the devulcanizing agent to mechano–chemically devulcanize ground tire rubber and then achieved in situ grafting of HDPE onto rubber chains.

In our previous work, we utilized TEPA as the devulcanizing agent to reclaim waste rubber and achieved superior performance in RRs [31]. However, the utilization of TEPA failed to reduce the Mooney viscosity of RRs and to enhance the devulcanizing efficiency. In another study, we used aminolysis products of waste polyurethane as devulcanizing agents and found that the aminolysis products could accelerate the devulcanization process and decrease the Mooney viscosity [32]. Since the aminolysis products contained both amine groups and hydroxyl groups, hydroxyl groups may be responsible for the decrease of Mooney viscosity and improvement of devulcanizing efficiency. Therefore, several alcohol–amine compounds, including hydroxyethyl ethylenediamine (AEEA), ethanolamine (ETA), and diethanol amine (DEA), are investigated to explore their effect on devulcanization. AEEA, ETA, and DEA are chosen in this work because they have different amount ratios of hydroxyl group to amine group in a single molecular chain. Through them, the role of hydroxyl content in the devulcanization process can be approximately determined.

To our knowledge, it is the first time that AEEA, ETA, and DEA have been used in the mechano–chemical devulcanization of waste rubber. AEEA and ETA have slight toxicity and corrosiveness and are widely used in industrial production. DEA is non-toxic and non-corrosive. Therefore, it is acceptable to use AEEA, ETA, and DEA in the rubber reclamation process since their usage is small. The amine groups in the alcohol–amine compounds are expected to promote the breaking of crosslinking bonds, while the hydroxyl groups are expected to decrease Mooney viscosity, improve plasticity, and increase devulcanization efficiency. To confirm this speculation, various characterizations and analyses are conducted to investigate the impact of these alcoholic amines on rubber devulcanization.

## 2. Materials and Methods

### 2.1. Materials

The rubber powder (RP) with 16-mesh sizes is produced from scrap truck tire tread, which was provided by Shandong Newdongyue Renewable Resources Technology Co., Ltd., Taian, China. The compositions of the RPs include NR, carbon black, zinc oxide, stearic acid, Bis[3-(triethoxysilyl)propyl]tetrasulfide, tear-resistant resin, N-1,3-dimethylbutyl-N′-phenyl-p-phenylenediamine, poly(1,2-dihydro-2,2,4-trimethyl-quinoline) (RD), wax, N-(Cyclohexylthio)phtalimide (CTP), sulfur, and N-cyclohexylbenzothiazole-2-sulphenamide (CZ), with the mass ratio of 100.0:12.0:45.0:3.5:2.0:2.0:5.0:2.0:1.5:1.0:0.2:1.0:1.25.

The additives used for rubber devulcanization include a devulcanizing agent and different alcohol–amine compounds. The alcohol–amine compounds include tetraethylenepentamine (TEPA, chemically pure, 99.5%, CAS: 112-57-2, Fuchen Chemical Reagent Co., Ltd., Tianjin, China), hydroxyethyl ethylenediamine (AEEA, chemically pure, 99%, CAS: 111-41-1, Guangzhou Jiangshun Chemical Technology Co., Ltd., Guangzhou, China), ethanolamine (ETA, chemically pure, 98%, CAS: 141-43-5, Shanghai Aladdin Biochemical Technology Co., Ltd., Shanghai, China), and diethanol amine (DEA, chemically pure, 98%, CAS: 111-42-2, Shanghai McLean Biochemical Technology Co., Ltd., Shanghai, China). The curing additives include zinc oxide (chemically pure, 99%), stearic acid (chemically pure, 99%), sulfur (chemically pure, 99%), microcrystalline wax (chemically pure, 99%), RD (chemically pure, 99%), N-tert-butylbenzothiazole-2-sulphenamide (NS) (chemically pure, 99%), and CTP (chemically pure, 99%), which were purchased from Sinopharm Chemical Reagents Co., Ltd., Shanghai, China.

**Figure 1:**
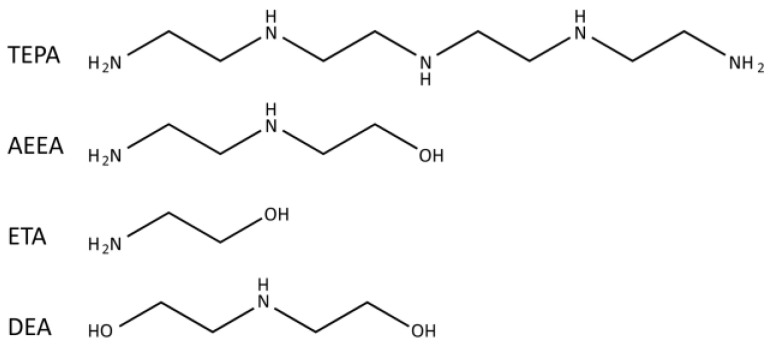
Molecular structures of the devulcanizing agents, including tetraethylenepentamine (TEPA), hydroxyethyl ethylenediamine (AEEA), ethanolamine (ETA), and diethanol amine (DEA).

The molecular structures of the considered devulcanizing agents are shown in Figure 1. Among them, TEPA has been used for the devulcanization of waste rubber in our previous work, and its appropriate dosage is 0.9 per hundred rubber (phr) based on comprehensive consideration of the tensile properties of vulcanized RRs. The amine group is the key group in the devulcanizing agent that promotes the decrosslinking process. To ensure the same number of amine groups, the dosage of different devulcanizing agents $w_{x}$ were calculated according to relative molecular mass and number of amine groups in each molecular chain as follows:

(1) $$w_{x}=w_{TEPA}\cdot \frac{M_{x}}{M_{TEPA}}$$

where $w_{TEPA}$ is the amount of TEPA, $M_{TEPA}$ is the molar mass of TEPA, $M_{x}$ is the molar mass of different devulcanizing agents. After calculations, 1.24 phr AEEA, 1.45 phr ETA, and 2.5 phr DEA were used in this work.

### 2.2. Experimental Procedure

#### 2.2.1. Devulcanization

The RPs were first mixed with the devulcanizing agents and alcohol–amine compounds in an internal mixer with a rotor speed of 50 rpm at 60 °C for 3 min. The rotors of the internal mixer are designed as shear-type synchronous rotors with six wings to enhance their shearing capacity. This design enables the plastication of RPs and facilitates the penetration of devulcanizing agents into them. Then, the RP compound was devulcanized on a refiner by stretching and shearing with a rotor speed of 25 rpm at the minimum roll gap until the RR completely sticks to the rollers and the devulcanization process cannot continue. As a result, there is a difference in the devulcanization time when different devulcanizing agents are used.

#### 2.2.2. Vulcanization of RRs

The recipe for curing the RRs is 100 phr RRs, 1.46 phr sulfur, 0.165 phr stearic acid, and 0.4 phr NS. The RR was first masticated on an open mill at a roller spacing of 0.2 mm twice. Then, the curing additives were added to the RR, and the mixture was mixed until it passed the roller 15 times. Afterward, the RRs were removed from the open mill and then put vertically into the open mill for additional mixing six times. Then, the curing characteristics of the obtained RR compound were tested to determine the optimum curing time.

For the curing recipe used in this work, the amounts of stearic acid and NS were decreased, and zinc oxide was not added because they are still partially or totally effective after the devulcanization process, as we discussed in previous work [22]. During the vulcanization process, zinc oxide and stearic acid activate the accelerator NS to form a more reactive intermediate product with sulfur, known as the accelerator polysulfide compound. This compound triggers the production of cross-linkable free radicals or ions in rubber molecular chains, leading to the formation of polysulfides within these chains. Additionally, the polysulfide compounds on rubber molecular chains combine with the free radicals or ions of the rubber molecular chains to facilitate cross-linking. Finally, through the cleavage and rearrangement of crosslinking bonds, a stable crosslinked network is formed. Generally, the more crosslinked bonds formed, the higher the crosslinking density, the stronger the tensile strength, and the higher the thermal stability.

### 2.3. Characterization

X-ray photoelectron spectroscopy (XPS) measurement was carried out using an X-ray photoelectron spectroscopy (AXIS Supra, Shimadzu Co., Kyoto, Japan) to evaluate the element’s contents and bonding states on the surface of RRs. Binding energies were corrected to the carbon 1 s peak located at 284.8 eV.

Fourier transform infrared reflection (FTIR) was tested using an Infrared Spectrum Analyzer (EQUINOX55, Bruker, Ettlingen, Germany) with the wavenumber from 600 cm^−1^ to 4000 cm^−1^. The tests were conducted using film samples of the sol component of RRs. The resolution was 4 cm^−1^, and the number of scans was 32 per spectrum.

Thermal gravimetric analysis (TGA) was conducted using a thermogravimetric analyzer (TG209, Netzsch Instruments Manufacturing Co., Ltd., Weimar, Germany). The test was conducted under nitrogen protection, with a nitrogen flow rate of 20 mL min^−1^. The rubber sample was raised from 40 °C to 800 °C at a rate of 20 °C min^−1^. The residual weight was calculated by dividing the real-time weight by the initial weight.

Viscoelasticity was tested on a rubber process analyzer (RPA) (RPA2000, Alpha Technologies, Inc., Wilmington, DE, USA). Analyses of strain scanning in the range of 0.28–40% were conducted at the frequency of 1 Hz and temperature of 60 °C.

Curing characteristics were tested using a rotorless rheometer (MDR2000, Alpha Corporation, Sterling, VA, USA) based on the standard of ASTM D5289-2007a [33]. The rubber samples were prepared to disc shapes with 5 g, and the testing temperatures were 150 °C. Rheologic curves and some important indexes, including maximum torque (MH), minimum torque (ML), scorch time (T10), and optimum cure time (T90), were obtained.

Mechanical properties of rubbers were tested using a tension tester (UT-2060, U-CAN Dynatex Inc., Taiwan, China) based on the standard of ASTM-D412. The thickness of the rubber samples used in the tests was 2 mm, and the tensile stretching speeds were 500 mm min^−1^. The hardness of rubbers was tested using a shore durometer (LX-A, Shanghai Liuling Instrument Factory, Shanghai, China) based on the standard of ASTM-D2240 [34].

Dynamic mechanical properties were tested using a dynamic mechanics analyzer (GaBOME-TER-150, GABO Qualimeter Testanlagen GmbH, Ahlden, Germany). The tensile mode was adopted during temperature scanning with the frequency of 10 Hz, a static strain of 5%, a static stress of 70 N, a dynamic strain of 0.25%, a dynamic stress of 60 N, the temperature range of −65~65 °C, and the heating rate of 2 °C min^−1^.

The number-average molecular weight (Mn) was tested by gel permeation chromatography (GPC) (GPC515-2410System, produced by Waters, Milford, MA, USA). The GPC analysis was performed with tetrahydrofuran as the mobile phase at the mobile velocity of 1 mL min^−1^ and temperature of 30 °C.

Sol content was tested using a Soxhlet extraction, and crosslink density was tested using the equilibrium swelling method with the Flory–Rehner equation [25]. The exact test process of sol content and crosslink density and the obtained Horikx curve based on them are consistent with our previous work [23].

Mooney viscosity was tested on a Mooney viscometer (M3810C, Beijing Huanfeng Chemical Industry, Beijing, China). According to the standards ASTM D 2084 [35] and ASTM D 1646 [36] (ML 100 °C (1 + 4) min), the rubber sample was preheated for 1 min and then tested with a rotor speed of 2 r min^−1^ at a temperature of 100 °C for 4 min.

The swelling rate of vulcanized rubber in different devulcanizing agents was tested to characterize the penetration capability of the devulcanizing agents into rubber. The vulcanized rubber samples were cut into sheets with the size of 2 × 2 cm^2^ and weighed as m1. Then, the samples were separately immersed in TEPA, AEEA, ETA, and DEA. The samples were weighed and recorded every other day. The rate of penetration of different devulcanizing agents in vulcanized rubbers can be reflected by calculating the growth rate of the sample mass.

The aging resistance was characterized by the retention of tensile strength and elongation at break before and after aging. The aging process was conducted in an oven at 100 °C for 72 h.

## 3. Results and Discussion

### 3.1. Mechanism Analysis

**Figure 2:**
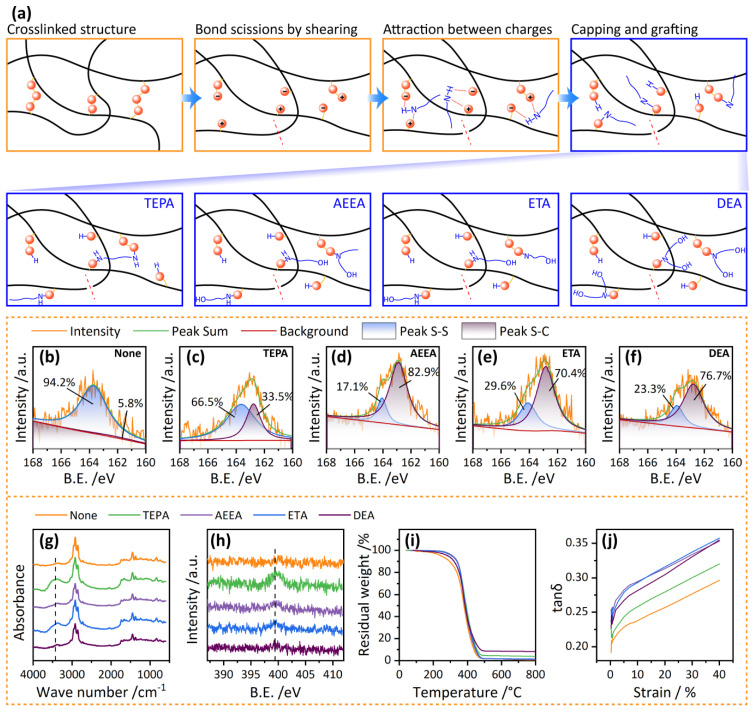
(**a**) Scheme of mechano–chemical devulcanization of waste rubbers using different devulcanizing agents. (**b**–**f**) X-ray photoelectron spectroscopy (XPS) results of S 2p for reclaimed rubbers (RRs) with no, tetraethylenepentamine (TEPA), hydroxyethyl ethylenediamine (AEEA), ethanolamine (ETA), and diethanol amine (DEA) as the devulcanizing agents; (**g**) Fourier transform infrared spectroscopy (FTIR) results; (**h**) X-ray photoelectron spectroscopy (XPS) results for N 1s; (**i**) variations of residual weight in thermogravimetric (TG) curves; and (**j**) damping factor as a function of strain in rubber process analyzer (RPA) results.

The molecular-scale devulcanization process using the amine-containing compounds as devulcanizing agents is depicted in Figure 2a. Vulcanized rubber molecules contain four types of chemical bonds, namely C=C, C-C, C-S, and S-S, with different bond energies (C=C > C-C > C-S > S-S). Due to the difference in bond energy, the mechanical shearing force can preferentially break S-S bonds. The cleavage of S-S bonds has two forms, one is homogeneous cleavage, producing two sulfur-free radicals, and the other is heterogeneous cleavage, producing positively charged -S^+^ and negatively charged -S^−^. When amine groups are present during the cleavage process of S-S bonds, their polarity will promote the S-S bond cleavage to turn towards heterogeneous cleavage, producing more -S^+^ and -S^−^. The outermost layer of amine groups has lone pair electrons, which have high reactivity and can capture positively charged -S^+^ and release H^+^. Free H^+^ will further bind with -S^−^ to form -SH terminal groups. At this point, the dissociation of the crosslink bonds has been completed, and the molecular chains are grafted with amine chains, thus blocking the activity of sulfur and preventing the re-crosslinking process. When TEPA is used as the devulcanizing agent, due to its multiple amine groups contained in a single molecular chain, one molecular chain of TEPA may be grafted into two or more rubber molecular chains, forming a complex structure, as shown in Figure 2a. When AEEA, ETA, or DEA is used as the devulcanizing agent, the grafting of amine groups introduces hydroxyl groups into the branched chains of rubber molecular chains. The specific molecular structures produced by using different devulcanizing agents may lead to some differences in the properties of RRs.

To confirm the devulcanization process, XPS, FTIR, and TGA were used to analyze the functional groups in the sol composition of RRs, and RPA was used to characterize the viscoelastic behavior. Figure 2b–f shows the typical narrow scan and curve-fit XPS results for S 2p. Two splits of S 2p peaks at 162.3 and 163.7 eV were assigned as the bonding energy for S-C and S-S bonds, respectively [37]. The proportion of peak area reveals that the S-S bond content in the RRs without adding any additives is quite high. After using TEPA, a notable decrease in the S-S bond content is observed. Similarly, when employing three types of alcohol amine additives, significant reductions in the S-S bond content are noted, illustrating that large amounts of S-S bonds are broken. The result fully demonstrates the promoting effect of alcoholic amines on breaking crosslinking bonds during the devulcanization process.

Figure 2g shows the FTIR results of RRs devulcanized with different additives. It shows an absorbance peak at around 3200–3600 cm^−1^, which corresponds to N-H groups. Without the use of a devulcanizing agent during the devulcanization process, there are no peaks observed in the sol composition of RR. However, when an amine or alcohol amine compound is used for the devulcanization, significant absorbance peaks are observed in the sol compositions of RRs, indicating that the molecular chains of the devulcanizing agents have successfully grafted onto the rubber chains and formed the sol compositions. When comparing TEPA, AEEA, ETA, and DEA as devulcanizing agents, it is observed that the N-H peaks in the TEPA and ETA curves are higher than those in the AEEA curve, while the N-H peak in the AEEA curve is more pronounced than in the DEA curve. This is because AEEA contains only secondary amine groups and no primary amine groups. When the secondary amine groups in AEEA react with –S^+^, the H^+^ is released from the secondary amine groups. As a result, the molecule chains in RRs have few N-H bonds after devulcanization. Therefore, the N-H peak in the FTIR of the RRs using AEEA as the devulcanizing agent is very weak. Similarly, the strength of the N-H peaks in RRs using other devulcanizing agents also depends on the type of amine groups present in the devulcanizing agents.

Figure 2h shows the XPS results of RRs for N 1s. The peak at around 400 eV represents the bonding energy for C-N bonds [38]. For RRs without using amine or alcoholic amines, the XPS result shows no peak at around 400 eV. The addition of amine or alcoholic amines resulted in the appearance of N 1s peak, and the peaks of TEPA and ETA were higher than those of AEEA and DEA. The regularity of this result is consistent with that of FTIR results, which further confirms that the amine groups have been successfully grafted onto the molecular chains of RRs.

In the TG results in Figure 2i, as the temperature exceeds 250 °C, the TG curves of the sol samples begin to differentiate. The weight loss rate of sol compositions without a devulcanizing agent is the fastest. The addition of devulcanizing agents slows down the degradation process. This may be attributed to the grafting of molecular chains from devulcanization agents, which form branching structures and improve the thermal stability of rubber samples. For sol compositions that used devulcanization agents during the devulcanization process, DEA showed a faster weight loss rate than other agents. This may be because DEA contains more hydroxyl groups, making it easier to decompose and produce H_2_O during temperature increases, thus accelerating weight loss.

In the RPA result in Figure 2j, the dumping factor of the RR without using the devulcanizing agents is lower than that using TEPA, and the dumping factor of the RR using TEPA is lower than those using AEEA, ETA, and DEA. The increase in the dumping factor after adding the devulcanizing agents is due to the breakage of crosslink bonds and the capping of the sulfur groups by the amine groups in the devulcanizing agent, which improves the mobility of the rubber chains. For the four cases using the devulcanizing agents, the TEPA case shows a lower dumping factor than the other cases. This may be because different amines in the same molecular chain of TEPA are grafted into different rubber molecular chains, forming a crosslinking structure and thus decreasing the dumping factor. The results of FTIR, TG, and RPA indicate that the amine groups in the devulcanizing agents can react with -S^+^ and -S^−^ and block their activity, finally introducing hydroxyl groups into the molecular chains of RRs.

### 3.2. Devulcanization Degree

**Figure 3:**
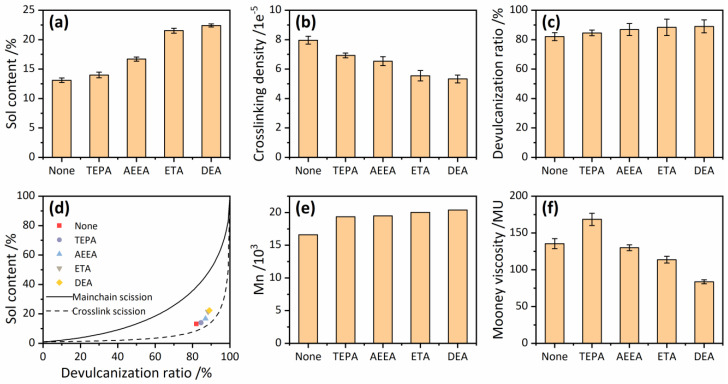
Sol content (**a**), crosslink density (**b**), devulcanization ratio (**c**), Horikx curves (**d**), number-average molecular weight (Mn) (**e**), and Mooney viscosity (**f**) of reclaimed rubbers (RRs).

The results of FTIR, TG, and RPA have demonstrated that the devulcanizing agents can seal off the activity of sulfur generated by the scissions of crosslink bonds. This outcome can hinder the re-crosslinking of -S^+^ and -S^−^, thereby affecting the devulcanizing degree of RRs. Sol content, gel crosslink density, and devulcanization ratio are intuitive parameters for determining the devulcanization degree of rubber, as shown in Figure 3a–c. Compared with RR with no devulcanizing agents, adding TEPA, AEEA, ETA, and DEA as the devulcanizing agents increased the sol content of RRs by 6.4%, 21.6%, 39.2%, and 41.5%, respectively, and the devulcanization ratio by 2.9%, 5.5%, 7.1%, and 7.8%, respectively, showing a significant promoting effect of devulcanization. The magnitude of sol content and devulcanization ratio are both DEA > ETA > AEEA > TEPA > None, and the magnitude of crosslink density is the opposite. This result indicates that the increase in hydroxyl groups of the devulcanizing agent is beneficial to the devulcanization of waste rubbers. The role of hydroxyl groups is mainly to increase the mobility of the devulcanizing agent. The devulcanizing agent with more hydroxyl groups can better disperse and penetrate the rubber matrix, thus promoting the devulcanization process. Additionally, when a molecular chain contains more than one amine group after one of the amine groups is grafted to the rubber molecular chain, it is difficult for other amine groups to be grafted to other rubber molecular chains. Therefore, under the premise that the total amount of amine groups is the same, the fewer amine groups in a single molecular chain have a better devulcanization effect. The order of the content of amine groups in a single molecular chain is TEAP > AEEA > ETA = DEA, so the devulcanization effect is increased sequentially.

Based on sol content and devulcanization ratio, Horikx curves were plotted as shown in Figure 3d. In the Horikx curves, the dashed line corresponds to more selective crosslink scissions, and the solid line corresponds to main-chain scissions and polymer degradation. Figure 3d shows that the devulcanization process in this work possesses high bond-breaking selectivity, and the addition of the devulcanizing agent has little effect on it. Additionally, it can be inferred from the Horikx theory that a lower sol content with a higher devulcanization degree corresponds to a higher bond-breaking selectivity. In the work of Azarabtin et al. [27], they obtained a sol content of 33.0% and a devulcanization ratio of 73.0% when using disulfide oil as the devulcanizing agent and the sol content of 37.0% and the devulcanization ratio of 80.0% when using tetramethyl thiuram disulfide as the devulcanizing agent. When using DEA as the devulcanizing agent in this work, the sol content and devulcanization ratio of the RR are 22.4% and 89.0%, respectively, indicating that the process possesses a higher devulcanization degree and a better bond-breaking selectivity.

Figure 3e illustrates the Mn values of the sol compositions for RRs. It is observed that the addition of devulcanizing agents enhances Mn. This can be attributed to two factors. Firstly, the infiltration of the agent into waste rubber allows for a more uniform distribution of mechanical forces within the rubber, resulting in improved selectivity of bond breaking and increased molecular weight of rubber molecular chains in the sol compositions. Secondly, the grafting of devulcanizing agents onto rubber molecular chains introduces hydroxyl groups, which may improve the solubility of RRs in toluene and thus increase Mn in the sol components of RRs. Therefore, an increase in Mn supports the conclusion that the devulcanization degree has been enhanced by the addition of devulcanizing agents.

Figure 3f displays the Mooney viscosity of rubber samples treated with different devulcanizing agents. It is observed that the addition of TEPA increased the Mooney viscosity while the addition of AEEA, ETA, and DEA reduced it. The increase in Mooney viscosity with TEPA may be due to the fact that different amine groups in the same molecular chain can be grafted onto different rubber molecular chains, forming a crosslinked structure with TEPA as the crosslink node. The decrease in Mooney viscosity with AEEA, ETA, and DEA is mainly attributed to the enhanced devulcanization degree. Additionally, alcoholic amines also have a plasticizing effect, which further reduces the Mooney viscosity of rubber samples. Therefore, when considering devulcanization effectiveness, alcohol–amine devulcanizing agents are superior to TEPA.

### 3.3. Devulcanizing Efficiency

**Figure 4:**
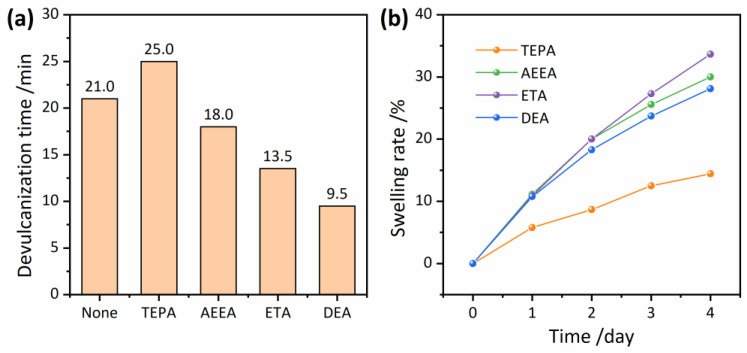
Devulcanization time with different devulcanizing agents (**a**) and variation of swelling rate of vulcanized rubbers over time (**b**).

The addition of alcohol–amine agents not only improves the devulcanization degree and reduces the Mooney viscosity of RRs, but also improves the production efficiency. As shown in Figure 4a, different devulcanizing agents have varying effects on the devulcanization time. TEPA prolonged the devulcanization time compared to the non-agent case, while AEEA, ETA, and DEA shortened it. The alcohol–amine agents showed the highest effect, with AEEA having the longest devulcanization time, followed by ETA and DEA. This indicates that increasing the hydroxyl content in the devulcanizing agents can significantly reduce the devulcanization time. In fact, the devulcanization time required for the process is reduced by around 31%, 38%, and 55%, respectively, when adding AEEA, ETA, and DEA compared to the non-agent case.

The reduction of devulcanization time using alcohol–amine devulcanizing agents is primarily attributed to their plasticizing effect, which enables them to rapidly disperse and penetrate the rubber matrix, thus accelerating the devulcanization process. To verify this hypothesis, swelling experiments were conducted on vulcanized NR with four different devulcanizing agents, and the results are shown in Figure 4b. It was observed that the swelling rate of the vulcanized NR in AEEA, ETA, and DEA was significantly higher than that in TEPA, indicating that alcohol–amine compounds have a higher impregnation rate into vulcanized NR than TEPA, allowing them to diffuse more quickly and speed up the devulcanization process. For the three alcohol–amine compounds, ETA had the highest infiltration efficiency, followed by AEEA and DEA.

From these results, it is evident that the efficiency of devulcanization is not always directly proportional to impregnation capacity. This is due to the fact that to ensure consistent numbers of amine groups, DEA has a higher addition amount than ETA, and ETA has a higher addition amount than AEEA. The higher the amount of agents added, the easier it becomes for the devulcanization process of RPs to reach the state of tightly adhering to the roll. It is worth noting that shortening the devulcanization time will not result in inadequate devulcanization, as shown in Figure 3, nor will it have a decisive countereffect on the performance of the RRs. The performance of RRs will be discussed in further detail in subsequent sections.

### 3.4. Properties of RRs

#### 3.4.1. Curing Characteristics

Table 1 presents the curing properties of RRs obtained using different devulcanizing agents. The value of ML is related to the mobility of RRs, with a higher ML value indicating better mobility. It was observed that the addition of devulcanizing agents reduced ML value and decreased further as the devulcanizing agents contained more hydroxyl groups. This result can be attributed to both the improved devulcanization degree, which restores the linear molecular chain structure of waste rubbers, and the introduction of hydroxyl groups into the rubber molecular chains, which may improve plasticity.

The value of ∆M is the difference between MH and ML, and it can reflect crosslink density in vulcanized RRs. It was found that the crosslink density of vulcanized RRs treated with devulcanizing agents during the devulcanization process was higher than that without adding a devulcanizing agent. This is due to the facilitation of the devulcanization process by amine groups in devulcanizing agents. For the cases using devulcanizing agents, the values of ∆M were TEPA > AEEA > ETA > DEA, indicating that the crosslink density decreases sequentially. This result may be due to the mass of devulcanizing agents sequentially increasing, which causes an increase in the number of small molecules in RRs and thus has a negative effect on crosslink density. The amine-containing devulcanizing agents also affected curing efficiency. It was observed that the addition of devulcanizing agents shortened the scorch time (T10) and optimum curing time (T90). This was due to the promotion of the vulcanization effect by amine groups in devulcanizing agents.

#### 3.4.2. Tensile Properties

**Figure 5:**
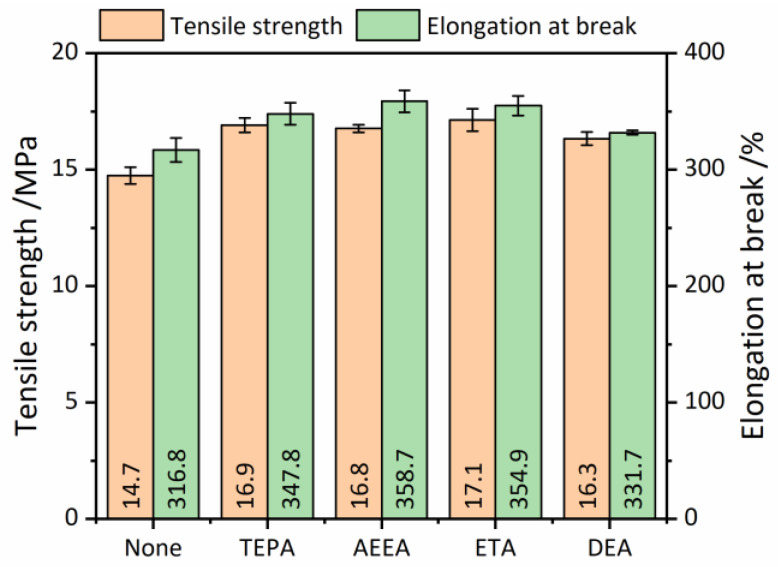
Tensile strength and elongation at break of the vulcanized reclaimed rubbers (RRs) with different devulcanizing agents.

Figure 5 shows the tensile strength and elongation at break of the vulcanized RRs obtained by using different devulcanizing agents. When no devulcanizing agent was added, the tensile strength of the RRs was 14.7 MPa, and the elongation at break was 316.8%. Adding the devulcanizing agent can enhance both the tensile strength and the elongation at break. The simultaneous increase in tensile strength and elongation at break is attributed to the devulcanization effect of the devulcanizing agents, which restores the crosslinked network structure of rubbers to a linear structure so that the molecular chains can be re-crosslinked with each other during the re-vulcanization process. Among different devulcanizing agents, ETA achieved the best tensile properties of RRs, that is, the tensile strength of 17.1 MPa and the elongation at break of 354.9%. This value of tensile strength is much higher than that found in other work, such as 12.90 MPa [39] and 11.00 MPa [21], which are relatively high tensile strengths for the RRs we found in the literature.

The results showed that the grafting of hydroxyl groups into the rubber molecular chain does not adversely affect the properties of the RRs. In addition, it is noted that the tensile strength and elongation at break of RR using DEA as the devulcanizing agent are relatively lower than those using other agents. This may be attributed to the excessive amount of DEA, which creates a certain spatial site resistance and weakens the crosslink between rubber molecular chains. As a result, the overall tensile properties are impacted.

#### 3.4.3. Anti-Aging Property

**Figure 6:**
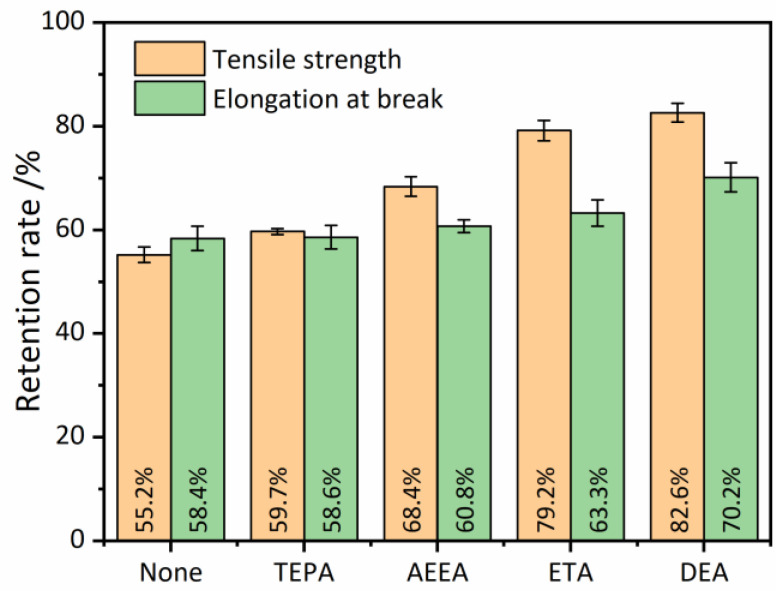
Anti-aging property of the reclaimed rubbers (RRs) reflected by variation of tensile strength and elongation at break.

The anti-aging properties of vulcanized RRs were characterized by comparing their tensile properties before and after aging, as shown in Figure 6. When no devulcanizing agent was added, the retention rates of the tensile strength and elongation at break of the vulcanized RR were 55.2% and 58.4%, respectively. The addition of TEPA slightly increased these retention rates to 59.7% and 58.6%, respectively. When alcohol–amine devulcanizing agents were added, the retention rates of both tensile strength and elongation at break were significantly improved. The best aging performance was achieved by adding DEA, which resulted in retention rates of 82.6% and 70.2%, respectively, for tensile strength and elongation at break. These results suggest that the anti-aging performance of RR can be improved with the increase in hydroxyl content, as evidenced by the introduction of hydroxyl groups. However, it is important to note that the effect of hydroxyl groups on aging performance has not been previously reported in the literature, and further research is needed to fully understand this phenomenon.

#### 3.4.4. Dynamic Mechanical Properties

**Figure 7:**
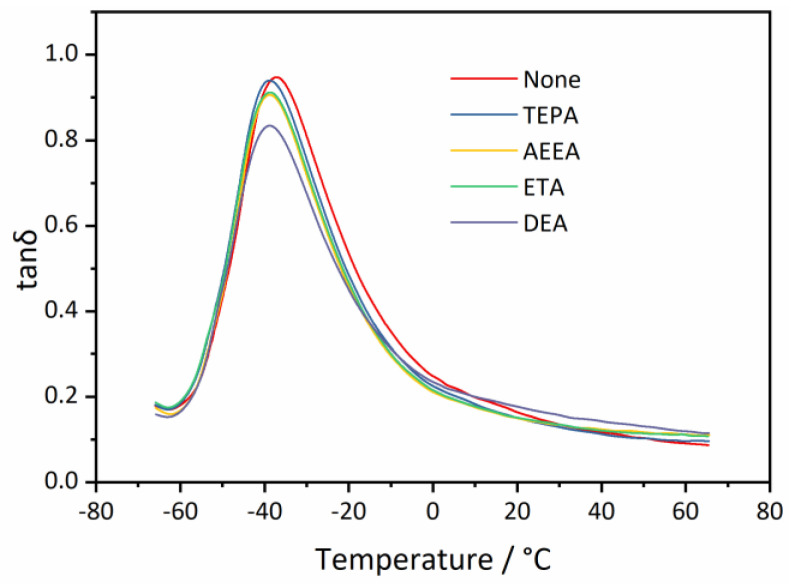
Temperature dependence of tanδ for vulcanized reclaimed rubbers (RRs) using different devulcanizing agents in DMA results.

DMA was performed to investigate the dynamic mechanical properties of vulcanized RRs treated with different devulcanizing agents. The results of DMA, which illustrate the relationships between tanδ and temperature, are shown in Figure 7. The temperatures at the peaks of the curves correspond to the glass transition temperature (Tg). As depicted in the figure, the Tg of an RR sample without a devulcanization agent is −37.1 °C. Upon addition of the devulcanizing agents, Tg is reduced to around −38.8 °C. This outcome suggests that the addition of devulcanization agents can enhance the low-temperature resistance of RRs. The variation in Tg may be linked to the branched chains present in rubber molecular chains. These branched chains have higher mobility than the main rubber chains, and their presence may impede the crystallization process of rubber molecular chains, thus reducing Tg. Additionally, the value of tanδ at 0 °C characterizes wet skid resistance, while the value of tanδ at 60 °C characterizes rolling resistance. It is observed that adding devulcanization agents has no significant effect on wet skid resistance or rolling resistance.

## 4. Conclusions

In this work, alcohol–amine compounds were utilized as devulcanizing agents in the low-temperature mechano–chemical devulcanization process of waste rubber to enhance the properties and efficiency of RR production. The results of FTIR, TG, and RPA analysis indicate that the amine groups in the devulcanizing agents can graft onto rubber molecular chains, forming branched chains and introducing hydroxyl groups into the rubber chains. Alcohol–amine compounds have superior devulcanization effects and efficiency due to the plasticizing effect of hydroxyl groups, which can improve the devulcanization degree, reduce the Mooney viscosity, and greatly accelerate the devulcanization rate. Additionally, the use of alcoholic amines can slightly improve tensile strength and elongation at break while substantially enhancing aging resistance. When DEA was used as the devulcanizing agent, the retention of tensile strength and elongation at break reached 82.6% and 68.7%, respectively, while no devulcanizing agent was used, resulting in retention levels of only 55.2% and 58.4%, respectively. The improvement in the aging resistance of RRs may be attributed to the increase in hydroxyl content in the devulcanizing agents, while the underlying mechanism remains unclear, and further research is needed. This work demonstrates the successful grafting of hydroxyl groups into rubber molecular chains during devulcanization, thereby improving the performance of RRs and providing a novel approach for reclaiming and modifying waste rubber, making it valuable for scientific advancement in this field.

## Figures and Tables

**Table 1:** Curing properties of reclaimed rubbers (RRs) obtained by using different devulcanizing agents.

Samples	MH/N m	ML/N m	ΔM/N m	T10/min	T90/min
None	14.67 ± 0.82	2.78 ± 0.19	11.89 ± 0.74	2.94 ± 0.21	5.68 ± 0.32
TEPA	15.78 ± 0.75	2.59 ± 0.15	13.29 ± 0.83	2.49 ± 0.16	4.30 ± 0.25
AEEA	15.64 ± 0.44	2.21 ± 0.13	13.21 ± 0.54	2.42 ± 0.18	3.59 ± 0.21
ETA	15.07 ± 0.66	2.14 ± 0.15	12.93 ± 0.80	2.44 ± 0.10	2.49 ± 0.27
DEA	14.48 ± 0.23	2.09 ± 0.09	12.39 ± 0.27	2.41 ± 0.16	3.84 ± 0.29

## Data Availability

Data will be available on request.

## References

[1] Abbas-Abadi M.S., Kusenberg M., Shirazi H.M., Goshayeshi B., Van Geem K.M. (2022). Towards full recyclability of end-of-life tires: Challenges and opportunities. J. Clean Prod..

[2] Advincula P.A., Luong D.X., Chen W., Raghuraman S., Shahsavari R., Tour J.M. (2021). Flash graphene from rubber waste. Carbon.

[3] Wisniewska P., Wang S.F., Formela K. (2022). Waste tire rubber devulcanization technologies: State-of-the-art, limitations and future perspectives. Waste Manag..

[4] Hittini W., Mourad A.-H.I., Abu-Jdayil B. (2021). Utilization of devulcanized waste rubber tire in development of heat insulation composite. J. Clean Prod..

[5] Hong Y.J., Jeong K.M., Saha P., Suh J., Kim J.K. (2015). Processing and characterization of microwave and ultrasonically treated waste-EPDM/LDPE polymer composites. Polym. Eng. Sci..

[6] Li X., Deng X.Q., Dong C. (2018). Effect of Temperature on Devulcanization of Waste Sidewall Rubber by Supercritical Ethanol. J. Braz. Chem. Soc..

[7] Chittella H., Yoon L.W., Ramarad S., Lai Z.W. (2021). Rubber waste management: A review on methods, mechanism, and prospects. Polym. Degrad. Stab..

[8] Zhao X.P., Hu H., Zhang D.K., Zhang Z., Peng S.X., Sun Y.M. (2019). Curing behaviors, mechanical properties, dynamic mechanical analysis and morphologies of natural rubber vulcanizates containing reclaimed rubber. e-Polymers.

[9] Sabzekar M., Zohuri G., Chenar M.P., Mortazavi S.M., Kariminejad M., Asadi S. (2016). A new approach for reclaiming of waste automotive EPDM rubber using waste oil. Polym. Degrad. Stab..

[10] Costamagna M., Brunella V., Luda M.P., Romagnolli U., Muscato B., Girotto M., Baricco M., Rizzi P. (2022). Environmental assessment of rubber recycling through an innovative thermo-mechanical devulcanization process using a co-rotating twin-screw extruder. J. Clean Prod..

[11] Sripornsawat B., Saiwari S., Pichaiyut S., Nakason C. (2016). Influence of ground tire rubber devulcanization conditions on properties of its thermoplastic vulcanizate blends with copolyester. Eur. Polym. J..

[12] Liu H.L., Wang X.P., Jia D.M. (2020). Recycling of waste rubber powder by mechano-chemical modification. J. Clean Prod..

[13] Formela K., Hejna A., Zedler L., Colom X., Canavate J. (2019). Microwave treatment in waste rubber recycling-recent advances and limitations. Express Polym. Lett..

[14] Vahdatbin M., Jamshidi M. (2022). Using chemical agent in microwave assisted devulcanization of NR/SBR blends: An effective recycling method. Resour. Conserv. Recy..

[15] Karabork F., Pehlivan E., Akdemir A. (2014). Characterization of styrene butadiene rubber and microwave devulcanized ground tire rubber composites. J. Polym. Eng..

[16] Molanorouzi M., Mohaved S.O. (2016). Reclaiming waste tire rubber by an irradiation technique. Polym. Degrad. Stab..

[17] Gumede J.I., Hlangothi B.G., Woolard C.D., Hlangothi S.P. (2022). Organic chemical devulcanization of rubber vulcanizates in supercritical carbon dioxide and associated less eco-unfriendly approaches: A review. Waste Manag. Res..

[18] Sabzekar M., Chenar M.P., Mortazavi S.M., Kariminejad M., Asadi S., Zohuri G. (2015). Influence of process variables on chemical devulcanization of sulfur-cured natural rubber. Polym. Degrad. Stab..

[19] Ghorai S., Bhunia S., Roy M., De D. (2016). Mechanochemical devulcanization of natural rubber vulcanizate by dual function disulfide chemicals. Polym. Degrad. Stab..

[20] El-Nemr K.F., Raslan H.A., Ali M.A.M., Hasan M.M. (2020). Innovative gamma rays irradiated styrene butadiene rubber/reclaimed waste tire rubber blends: A comparative study using mechano-chemical and microwave devulcanizing methods. J. Polym. Eng..

[21] Seghar S., Asaro L., Rolland-Monnet M., Hocine N.A. (2019). Thermo-mechanical devulcanization and recycling of rubber industry waste. Resour. Conserv. Recycl..

[22] Guo L., Lv D., Ren D., Qu L., Wang W., Hao K., Guo X., Chen T., Sun J., Wang C. (2021). Effectiveness of original additives in waste rubbers for revulcanization after reclamation with a low-temperature mechanochemical devulcanization method. J. Clean Prod..

[23] Guo L., Wang C., Lv D., Ren D., Zhai T., Sun C., Liu H. (2021). Rubber reclamation with high bond-breaking selectivity using a low-temperature mechano-chemical devulcanization method. J. Clean Prod..

[24] Gagol M., Boczkaj G., Haponiuk J., Formela K. (2015). Investigation of volatile low molecular weight compounds formed during continuous reclaiming of ground tire rubber. Polym. Degrad. Stab..

[25] Shi J., Jiang K., Ren D., Zou H., Wang Y., Lv X., Zhang L. (2013). Structure and performance of reclaimed rubber obtained by different methods. J. Appl. Polym. Sci..

[26] Ghosh J., Ghorai S., Bhunia S., Roy M., De D. (2018). The Role of Devulcanizing Agent for Mechanochemical Devulcanization of Styrene Butadiene Rubber Vulcanizate. Polym. Eng. Sci..

[27] Azarabtin S., Mousavi S.R., Khameneh R.J., Mortazavi S.M.M., Ehsani M., Ranjbar H., Khonakdar H.A. (2022). Effect of different devulcanization agents on the mechano-chemical devulcanization process of waste tires. Mater. Today Commun..

[28] Bockstal L., Berchem T., Schmetz Q., Richel A. (2019). Devulcanisation and reclaiming of tires and rubber by physical and chemical processes: A review. J. Clean Prod..

[29] Kojima M., Ogawa K., Mizoshima H., Tosaka M., Kohjiya S., Ikeda Y. (2003). Devulcanization of sulfur-cured isoprene rubber in supercritical carbon dioxide. Rubber Chem. Technol..

[30] Jiang C., Zhang Y.S., Ma L., Zhou L., He H. (2018). Tailoring the properties of ground tire rubber/high-density polyethylene blends by combining surface devulcanization and in-situ grafting technology. Mater. Chem. Phys..

[31] Guo L., Ren D., Wang W., Hao K., Guo X., Liu F., Xu Y., Liu M., Liu H. (2021). Low-Temperature Mechano-Chemical Rubber Reclamation Using Terpinene as a Swelling Agent to Enhance Bond-Breaking Selectivity. Polymers.

[32] Wang W., Hao K., Guo X., Liu F., Xu Y., Guo S., Bai L., Liu G., Qu L., Liu M. (2023). Mechano-chemical rubber reclamation using aminolysis products of waste flexible polyurethane foams as the devulcanizing agent. J. Clean Prod..

[33] (2007). Standard Test Method for Rubber Property-Vulcanization Using Rotorless Cure Meters.

[34] (2021). Standard Test Method for Rubber Property-Durometer Hardness.

[35] (2019). Standard Test Method for Rubber Property-Vulcanization Using Oscillating Disk Cure Meter.

[36] (2019). Standard Test Methods for Rubber—Viscosity, Stress Relaxation, and Pre-Vulcanization Characteristics (Mooney Viscometer).

[37] Li Y., Zhao S., Wang Y. (2012). Improvement of the properties of natural rubber/ground tire rubber composites through biological desulfurization of GTR. J. Polym. Res..

[38] Wagner A.J., Wolfe G.M., Fairbrother D.H. (2003). Reactivity of vapor-deposited metal atoms with nitrogen-containing polymers and organic surfaces studied by in situ XPS. Appl. Surf. Sci..

[39] Tao G.L., He Q.H., Xia Y.P., Jia G.C., Yang H.C., Ma W.Z. (2013). The effect of devulcanization level on mechanical properties of reclaimed rubber by thermal-mechanical shearing devulcanization. J. Appl. Polym. Sci..

